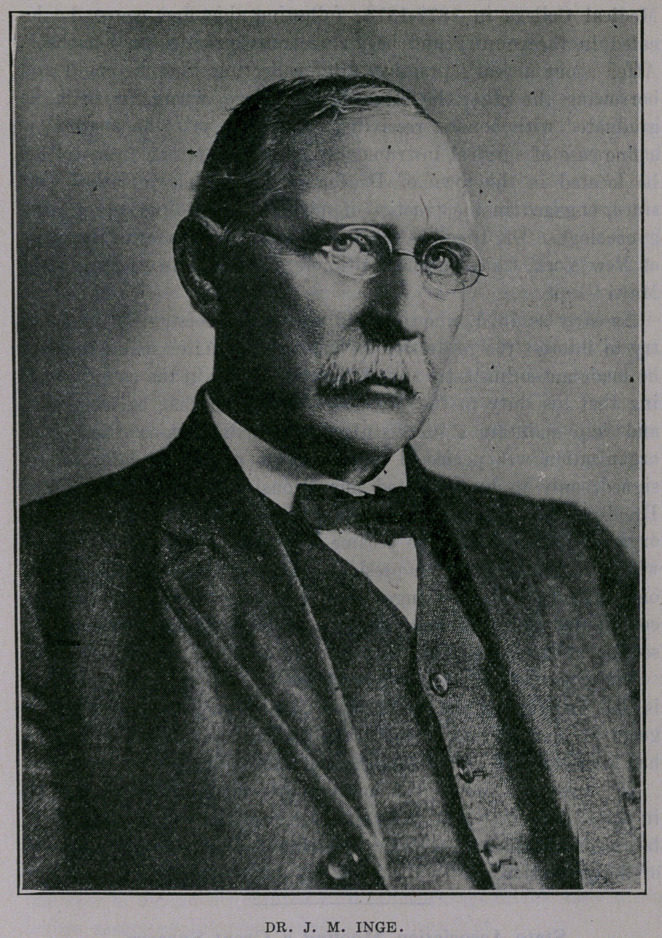# Some of Our Plans for the Future

**Published:** 1915-06

**Authors:** 


### EDITORIAL DEPARTMENT.

MRS. F. E. DANIEL, Publisher and Managing Editor.
DR. R. H. L. BIBB, Associate Editor.

CONTRIBUTING EDITORS:

Dr. H. Sheridan Baketel, New York, editor *Medical Times:* Dr. M. H. Boerner' Austin, Texas; Dr. G. Henri Bogart, Paris, Ill.; Dr. M. M. Carrick, Dallas, Texas; Dr. Edward H. Carey, Dean Medical Department, Baylor University, Dallas, Texas; Dr. W. L. Crosthwaite, Waco, Texas; Dr. T. D. Crothers, Hartford, Conn.; Dr. H. W. Cummings, Hearne, Texas; Dr. Oscar Dowling, New Orleans, President Louisiana State Board of Health; Havelock Ellis, M. D., London, Eng.; Dr. May Agnes Hopkins, Dallas, Texas; Dr. A. W. Fly, Galveston, Texas; Dr. G. B. Foscue, Waco, Texas; Dr. E. S. Goodhue, Holualoa, Kona, Hawaii; Dr. M. B. Grace, Seguin, Texas; Dr. Joe Gilbert. Austin, Texas; Dr. Charles H. Hughes, St. Louis, editor *Alienist and Neurologist*; Dr. James G. Kiernan, Chicago; Dr. George H. Lee, Galveston, Texas; Dr. John T. Moore, Houston, Texas; Dr. Frank Paschai, San Antonio, Texas; Dr. John Preston, Austin, Texas, Superintendent State Lunatic Asylum; Dr. G. Wilse Robinson, Kansas City, Mo.; Dr. Bacon Saunders, Fort Worth, Texas; Dr. Allen J. Smith, Philadelphia, Medical Department, University of Pennsylvania; Dr. Z. T. Scott, Austin, Texas; Dr. Ralph Steiner, Austin, Texas, President Texas State Board of Health; Dr. Walter S. Sutton, Kansas City, Mo.; Dr. A. E. Sweatland, Nacogdoches, Texas; Dr. John S. Turner, Dallas, Texas; Dr. Charles Venable, San Antonio, Texas.

---

### Some of Our Plans For the Future.

It is just one year ago this month since the writer assumed the entire responsibility of publishing the TEXAS MEDICAL JOURNAL, fully realizing at the time the enormity of the undertaking, but accepting the responsibility as cheerfully and as hopefully as possible. This was undertaken with no idea at the time of the wonderful possibilities for service to the profession that now so clearly appear.

Day and night during the past year I have labored lovingly and untiringly to give you, my readers and friends, the very best and most helpful articles and suggestions that could possibly be obtained. The JOURNAL has not in any sense of the word been filled with the ideas of any one person; on the contrary, it has contained the ideas of men who are big and broad; the men who are forging ahead; the men who are doing things. The editor's one thought and prayer has been that the influence of the JOURNAL should be for good. That those who have given their valiant support should receive far more than their investment called for. An earnest effort is being made to give you twelve dollars worth of valuable information for every dollar you invest in subscription.

I believe that I shall be able to do this during the coming year and am planning some great improvements. These ideas have come so fast that it has been impossible to put them into execution all at one time. Henceforth the insignia of the JOURNAL shall be service with all the word implies, not forgetting our last year's motto of Progress, Originality, Helpfulness.

Every page from the front cover to the back cover shall spell service; to this end, more and more effort will be made to see that every advertisement shall be clean, ethical, and what it purports to be. The TEXAS MEDICAL JOURNAL shall earnestly co-operate in every way with all high grade journals in bringing about a higher standard of ideals, morals and ethics among medical men, and in striving to raise the educational requirements of those seeking to enter the profession.

The JOURNAL'S friends and supporters are among the best men in the profession: nothing shall knowingly appear in this JOURNAL, in its advertising pages, or elsewhere, that might prove ethically offensive to them. Their able co-operation is too much appreciated for that. Some of them have been kind enough to point out that we are carrying advertisements at present that are not up to the standard; these will be dropped just as soon as the contracts expire. To give service honest advertising is just as essential as honest reading matter. Perhaps we feel more keenly on this subject than others do, but the friends who came to us in our sorrow and weakness and offered their sympathy and co-operation and whose very confidence was inspiration for planning and working constantly for a bigger and better TEXAS MEDICAL JOURNAL, is the dearest and most valued thing that we possess, and surely nothing shall be done to forfeit this friendship and esteem. So the statement is emphasized that the JOURNAL expects to rid its pages, just as rapidly as possible, of anything that is not what it is represented to be.

Plans have been made for a number of special issues during the coming year, which will be edited by men who are authorities on the subjects which they will handle. Dr. John T. Moore of Houston, Texas, will have charge of the July issue, which issue will be devoted to the study of the cancer problem.

Dr. Marvin L. Graves will have charge of an issue on the negro problem as it affects the health of the white race. There will be several other special issues, of which you will be informed from time to time.

The Package Library has been established, and this year it will be free to every paid up subscriber who wishes to take advantage of it. Already calls are being made for material relating to various subjects, and congratulations on this forward step are being received from the most thoughtful and earnest men in the profession. Something like five hundred dollars worth of the best journals published come to our desk each month. The articles in these are clipped and classified, so that at a moment's notice information on any subject can be sent forward to those asking for the same.

The Eugenics Department, under the supervision of Dr. Malone Duggan, will be continued. Dr. Duggan has given twenty years to the study of this subject and his department has received favorable comment both at home and abroad. Readers who are trying to keep well abreast of the times know full well the value of this department.

There are other plans for the betterment of the JOURNAL, but these will keep until some other time. The editor takes this occasion to thank every one who has contributed to the betterment of the JOURNAL through the past year and to ask your continued help and good will.

Attention is directed to the advertisement of the Westbrooke Hotel, of Fort Worth, which appears in this issue of the JOURNAL.

Mr. H. B. Christian, the president and manager, has succeeded in his desire to create a "clean," "homey" atmosphere, and as a host he has no peer.

Don't forget to stop there when you go to Fort Worth. Remember it is headquarters for visiting physicians, and if any of your doctor friends are in Fort Worth you will find them at the Westbrooke.

---

DEAF CHILDREN.—Anyone interested in a little deaf child can obtain free literature explaining approved methods of training deaf children from infancy to school age by writing to The Volta Bureau for the Increase of Diffusion in Knowledge Relating to the Deaf, 1601 Thirty-fifth Street, N. W., Washington, D. C. This literature relates only to the training of little deaf children; not to medical treatment nor to the deafness that come in later life. Age of child and other details are welcomed.

---

Dr. J. H. Gambrell is authority for the statement that plans to make Dallas the leading medical center of the South in connection with the Baptist Memorial Sanitarium and Baylor University College of Medicine will be launched soon by Dallas and Texas Baptists.

---

Dr. C. B. Jones of Quanah, formerly of New York, is now located at Paducah.

DR. GEO. H. MOODY.

Dr. Geo. H. Moody, the new President of the State Medical Association, was born in Mexia, Texas, May 12, 1872. He attended the public schools of Limestone county, and later the Southwestern University at Georgetown, Texas. He graduated in medicine from Tulane University, New Orleans, La., in 1896. After his return from college he practised medicine for a short time at his old home. He served as assistant physician of the State Insane Asylum at Austin, later serving four years as first assistant superintendent of the Southwestern Insane Asylum at San Antonio. He resigned from this position May 1, 1903, in order to continue his studies in nervous diseases and psychiatry in Europe. After his return from Europe he established "Moody's Sanitarium" at San Antonio for the treatment of nervous and mental diseases, of which he is now superintendent. For the past seventeen years his practice has been limited exclusively to nervous and mental diseases; during this time he has done post-graduate work in the New York Post-Graduate Medical School, New York Neurological Institute, and Bellvue Psychopathic Hospital. Dr. Moody has been a very active member of the medical societies, not only of his own country, but of the American Medico-Psychological Association and the Medical Association of the Southwest, of which he has been president. He has also served on the San Antonio Board of Health.

Dr. Moody believes that physicians should take part in other things than those relating to medicine, as evidenced by the fact that he is a Knight Templar Mason, a Shriner, an Elk and a Rotarian.

In 1907 he married one of San Antonio's most gracious and charming girls, Miss Bebe Denman. They have two splendid manly little boys, Geo. H., Jr., and Leroy Denman.

The Association did well in selecting Dr. Moody as its head for the coming year, for besides being a leader in his chosen profession, he is a good business man and keeps himself well informed on all subjects.

As a physician he has very high ideals, having as a boy been associated very closely with a physician of the old school, who felt that the practice of medicine was a calling, a sacred and holy calling. Dr. Moody must early have learned the Hippocratic obligation which begins with, "I do solemnly swear, by whatever I hold most sacred, that I will be loyal to the profession of medicine and just and generous to its members," for while regretting the fact that commercialism has crept into his profession, Dr. Moody is charitable and always ready to speak a kind word and lend a helping hand to those who may not have been so fortunate as he. This kindness, this feeling of brotherly love for all has made him a general favorite, and we earnestly hope and confidently predict that this will be a great year for the State Association.

DR. J. M. INGE.

Dr. J. M. Inge, the youngest of a family of nine children, was born in Kentucky, where he lived until six years of age. After the death of his father, a physician, the mother with her family moved to Fannin county, Texas, where he assisted in the farm work until sixteen years of age. After attending and getting all he could out of the country schools, he accepted a position as drug clerk in the only drug store then in the town of Denton, where he began the study of medicine. After two and one-half years of faithful and diligent study of anatomy, he attended the Louisville Medical College in 1872-1873; following this first course he located in the country and began a county practice on horseback. After about a year's practice, after collecting what he could and borrowing the other, he returned to college, where, in 1874, he graduated with honors, receiving Dr. Kelly's prize on anatomy—a fine case of surgical instruments. After his return from college he located in the town of Denton, where he has remained ever since, engaged in the practice of medicine, including surgery and gynecology. Dr. Inge has taken post-graduate courses in the cities of New York, Chicago, and Baltimore, his last course being in the Mayo Clinic.

As early as 1875, when we had no county societies, he began to try to interest the profession in such work. After many failures, he made an enthusiastic appeal to every doctor in the county, stating that his duty to the profession demanded that he meet with and help maintain a successful county medical association. The organization was perfected and he made president; later he resigned, only to be re-elected unanimously. Much credit is due Dr. Inge for his untiring efforts to build up and maintain this organization. In 1882 he became a member of the association of which he has been chosen president-elect, having been absent only one time from its meetings since becoming a member. He has contributed many valuable papers to the surgical and gynecological section of these meetings.

The consensus of the opinion of the profession in North Texas is that Dr. Inge is one of the most competent physicians and surgeons in that section, notwithstanding the fact that his practice has been confined to the country and country homes.

Dr. Inge has proved conclusively that one may attain success in spite of poverty and unfavorable environment; his life and attainment should be especially inspiring for the younger members of the profession.

## Figures and Tables

**Figure f1:**
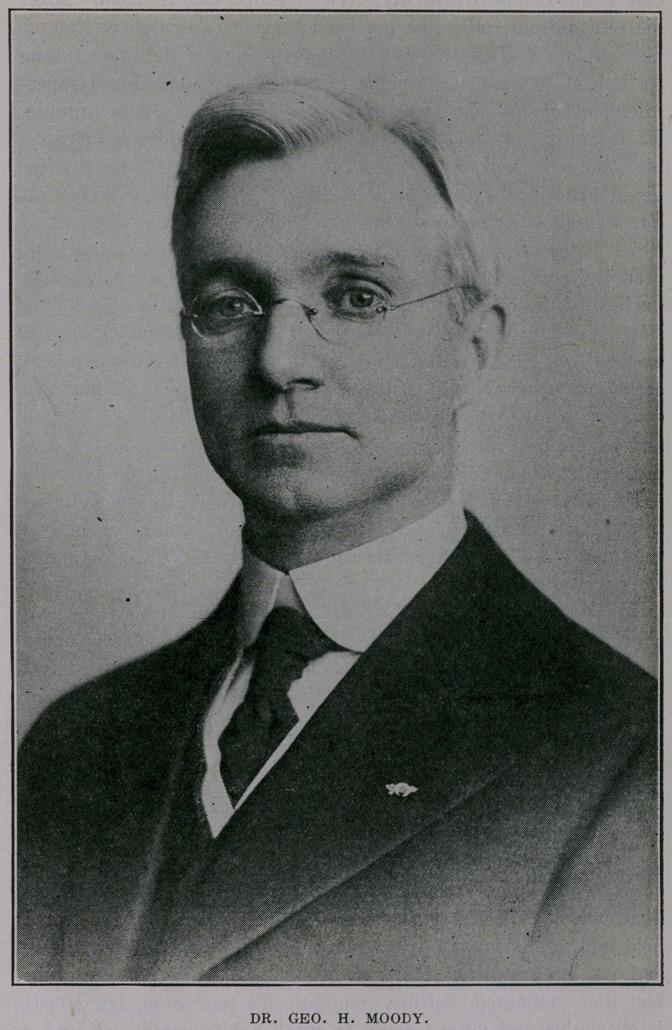


**Figure f2:**